# Crystal structure of posnjakite formed in the first crystal water-cooling line of the ANSTO Melbourne Australian Synchrotron MX1 Double Crystal Monochromator

**DOI:** 10.1107/S2056989020008099

**Published:** 2020-06-26

**Authors:** Stuart Mills, Jun Aishima, David Aragao, Tom Tudor Caradoc-Davies, Nathan Cowieson, Christine L. Gee, Daniel Ericsson, Stephen Harrop, Santosh Panjikar, Kate Mary Louise Smith, Alan Riboldi-Tunnicliffe, Rachel Williamson, Jason Roy Price

**Affiliations:** a Geosciences, Museum Victoria, GPO Box 666, Melbourne, Victoria, 3001, Australia; b Brookhaven National Laboratory, 743 Brookhaven Avenue, Upton, NY, USA; c Australian Synchrotron, ANSTO - Melbourne, 800 Blackburn Rd, Clayton, VIC, 3168, Australia; d Diamond Light Source, Diamond Light Source Ltd, Didcot, Oxfordshire, OX11 0DE, UK; e Department of Molecular and Cell Biology, University of California, Berkeley, CA 94720, USA

**Keywords:** crystal structure, copper corrosion, equipment failure, posnjakite, hydrogen bonding

## Abstract

Exceptionally large crystals of posnjakite, Cu_4_SO_4_(OH)_6_(H_2_O), formed during corrosion of a Swagelock(tm) Snubber copper gasket within the MX1 beamline at the ANSTO-Melbourne, Australian Synchrotron. The crystal structure was solved using synchrotron radiation to *R*_1_ = 0.029 and revealed a structure based upon [Cu_4_(OH)_6_(H_2_O)O] sheets, which contain Jahn–Teller-distorted Cu octa­hedra. The sulfate tetra­hedra are bonded to one side of the sheet *via* corner sharing and linked to successive sheets *via* extensive hydrogen bonds. The sulfate tetra­hedra are split and rotated, which enables additional hydrogen bonds.

## Abstract 2

Exceptionally large crystals of posnjakite, Cu_4_SO_4_(OH)_6_(H_2_O), formed during corrosion of a Swagelock(tm) Snubber copper gasket within the MX1 beamline at the ANSTO-Melbourne, Australian Synchrotron. The crystal structure was solved using synchrotron radiation and revealed a structure based upon [Cu_4_(OH)_6_(H_2_O)O] sheets, which contain Jahn–Teller-distorted Cu octa­hedra.

## Chemical context   

The MX1 beamline at the ANSTO-Melbourne Australian Synchrotron has been operating since 2007 (Cowieson *et al.*, 2015 ▸). The vacuum vessel for the double crystal monochromator suffered a loss of vacuum in July 2019. Investigation lead to the discovery of a pinhole leak in the water-cooling system for the first crystal. On disassembly, there was the discovery of corrosion, oxidation and a crust of crystal aggregates deposited on the Swagelock^(tm)^ Snubber copper gasket within the connection. The coolant for the system is reverse osmosis (RO) water, kept at constant temperature of 20°C by a Huber Chiller (CC-K6, Pilot ONE) and previously had Aqua-Stabil (Jubalo GmbH) added to prevent microbial and fungal growth. The lines are a combination of plastic and stainless steel, with water passing through an oxygen-free copper block into which the Silicon 111 first crystal is clamped with a layer of indium between the silicon crystal and the copper cooling block. The Aqua-Stabil was removed from service following a review of the environmental risks from chemicals in use. The pH-neutral RO water has been used as a coolant since 2015. In order to understand how the crystalline material was formed, the MX1 beamline was used after repairs were enacted.

**Figure 1:**
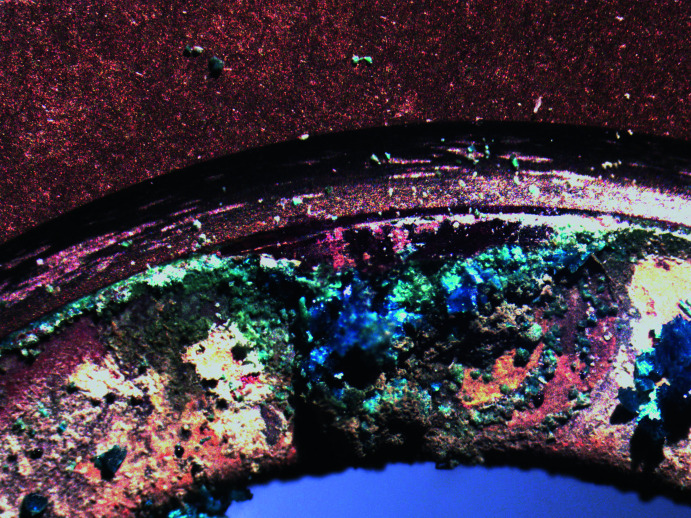
The copper Swagelok Snubber gasket from the MX1 cooling line for the first crystal in the double crystal monochromater showing crystalline deposits.

A thin film of red copper oxide (cuprite) coats the surface of the gasket, and on top of that sits a mat of crystals up to 0.5 cm thick (Fig. 1 ▸). Above the cuprite coating a pale-blue X-ray amorphous phase is observed, and perched on this phase sits exceptionally well-crystallized dark-blue crystals of posnjakite. The crystals of posnjakite are tabular on (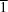
01) with maximum dimension of ∼0.2 × 0.2 × 0.2 mm. On some crystals, dark-green tips are observed where the crystals are transforming to brochantite.

## Formation of the crystals   

The corrosion of copper is of great inter­est in corrosion science, the arts and in the understanding of electrical apparatus failures. As is in our case, the copper corrosion has resulted in equipment failure. It has been seen that corrosive sulfur in oil has become a problem in electrical apparatus failures, where it is noted ‘*a number of failures of very large power transformers and shunt reactors associated with corrosive sulfur in electrical insulating mineral oils. Although the number of failures has been relatively small, perhaps 100 or so units, the assets lost have been substantial*’ (Griffin & Lewand 2007 ▸). Although no sulfur is in the MX1 system, the copper gasket was produced using milling oils that have sulfurized fat as a component. Even though these gaskets are cleaned before use, it is apparent that some residue remained and that it contains enough sulfur to produce abundant mineralization if a pinhole leak exists.

The production of copper patinas in several outdoor conditions has been charted (Krätschmer *et al.* 2002 ▸) and it was shown that cuprite was formed immediately, followed by an amorphous copper sulfate over hours to weeks, posnjakite over weeks to months and finally brochantite over years. We see this entire assemblage on the MX1 gasket; however, brochantite is the least common of the phases. We can use this data to estimate that the leak was present for approximately a year before failure. What is inter­esting is that the formation in this moderately closed system has enhanced the formation of posnajkite, creating sub-millimetre-sized crystals. It is worth noting that posnajkite is metastable with respect to brochantite (Zittlau *et al.*, 2013 ▸); however, it is also common especially in geological systems that the crystalline phase that may form in a system that is not the most stable one, but very frequently metastable with a simpler structure (Krivovichev, 2017 ▸).

## Structural commentary and supramolecular features   

**Figure 2:**
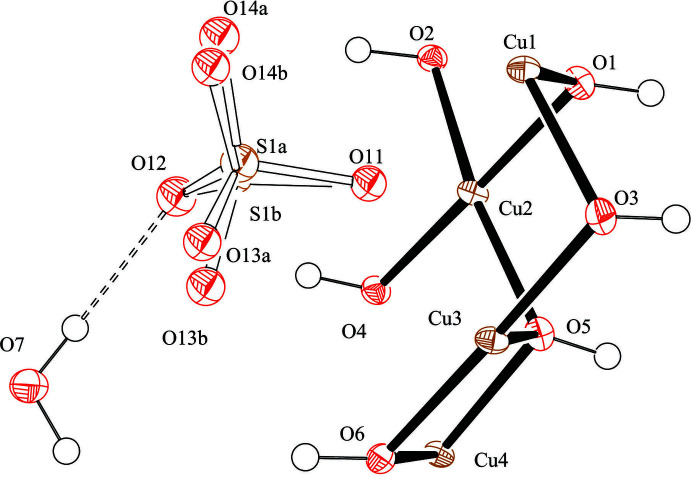
The asymmetric unit of posnjakite, the atomic numbering scheme is shown, and displacement ellipsoids are drawn at the 50% probability level.

Mellini & Merlino (1979 ▸) published the original structure of posnjakite, but with one H atom missing and limited thermal parameters. Our dataset has enabled the location of all H atoms from the difference map as well as an anisotropic model of all non-hydrogen atoms, except for the disordered sulfate. The posnjakite structure is based upon [Cu_4_(OH)_6_(H_2_O)O] sheets, which contain Jahn–Teller-distorted Cu octa­hedra. The average bond lengths for the octa­hedra are 2.08, 2.07, 2.11 and 2.11 for Cu1–4, respectively. These octa­hedra are more regular than the ones observed previously (Mellini & Merlino, 1979 ▸) because of the higher quality dataset and lower temperature refinement, which places the water mol­ecule ∼0.3 Å closer to the Cu atoms. The sheet is also less undulating than reported for the previous refinement. The asymmetric unit for the current structure is shown in Fig. 2 ▸.

**Figure 3:**
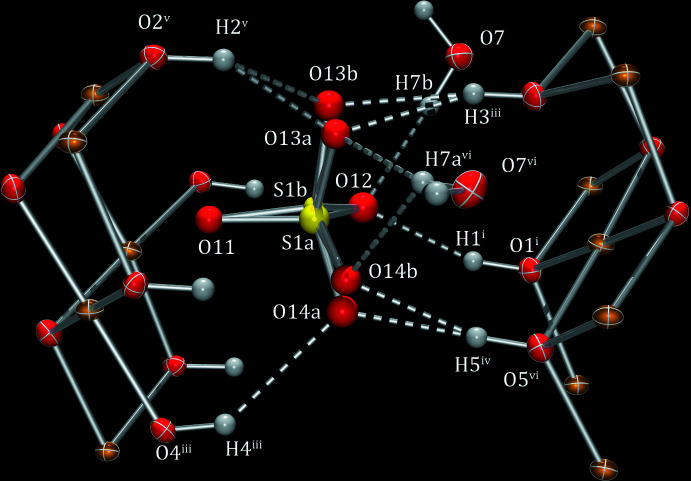
The complex arrangement of hydrogen bonds surrounding the disordered sulfate anion (*ORTEP-3*; Farrugia, 2012 ▸). Symmetry codes: (i) *x* + 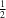, 1 − *y*, *z* − 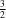; (ii) *x* + 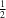, 2 − *y*, *z* + 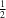; (iii) *x* + 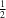, 2 − *y*, *z* − 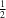; (iv) *x* + 1, *y*, *z*; (v) *x*, *y* + 1, *z*; (vi) *x* + 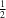, 2 − *y*, *z* + 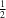.

The sulfate tetra­hedra are bonded to one side of the sheet *via* corner sharing and linked to successive sheets *via* extensive hydrogen bonds (Table 1 ▸). The main difference in this dataset is that the sulfate tetra­hedra are split and rotated by ∼7°. This allows greater connectivity between the sulfate oxygen atoms and the hydroxyl atoms in the sheet (*e.g.* O14*A*—H4) as well as the water of solvation (O13*A*—H7*A*, Fig. 3 ▸).

The tetra­hedra were restrained to have the grand mean <S—O> of 1.473 Å reported (Hawthorne *et al.* 2000 ▸), and the atoms kept isotropic.

## Refinement   

Crystal data, data collection and structure refinement details are summarized in Table 2 ▸. All non-hydrogen atom sites in the asymmetric unit were modelled with anisotropic displacement parameters with exception of the partially occupancy atoms which were left isotropic. The disordered sulfate tetra­hedra were restrained to have the grand mean <S—O> of 1.473 Å reported (Hawthorne *et al.* 2000 ▸), and the atoms kept isotropic. Hydrogen atoms were located in the difference map and their coordinates refined with group displacement parameters *U*_iso_(H) = 1.5*U*_eq_(O). Twinning of the crystal was explored to examine if the apparent disorder of the sulfate anion was from a twin component. An inversion twining was ruled out as the Flack (1983 ▸) parameter was 0.08 (4); further twinning was explored with TwinRotMat in the *PLATON* suite (Spek, 2020 ▸) with no twins found.

## Supplementary Material

Crystal structure: contains datablock(s) I. DOI: 10.1107/S2056989020008099/tx2024sup1.cif

Structure factors: contains datablock(s) I. DOI: 10.1107/S2056989020008099/tx2024Isup2.hkl

CCDC reference: 2010348

Additional supporting information:  crystallographic information; 3D view; checkCIF report

## Figures and Tables

**Table 1:** Hydrogen-bond geometry (Å, °)

*D*—H⋯*A*	*D*—H	H⋯*A*	*D*⋯*A*	*D*—H⋯*A*
O1—H1⋯O12^i^	0.87 (6)	1.86 (6)	2.693 (9)	160 (7)
O7—H7*B*⋯O12	0.87 (3)	2.02 (4)	2.844 (7)	158 (7)
O2—H2⋯O13*A* ^ii^	0.91 (6)	1.95 (6)	2.746 (9)	144 (7)
O2—H2⋯O13*B* ^ii^	0.91 (6)	1.96 (7)	2.731 (18)	141 (7)
O3—H3⋯O13*A* ^iii^	0.83 (6)	1.96 (6)	2.757 (10)	161 (9)
O3—H3⋯O13*B* ^iii^	0.83 (6)	1.99 (7)	2.799 (19)	164 (9)
O7—H7*A*⋯O13*A* ^iv^	0.89 (3)	2.13 (3)	2.995 (10)	167 (7)
O4—H4⋯O14*A* ^v^	0.86 (6)	2.22 (7)	2.929 (9)	141 (7)
O4—H4⋯O14*B* ^v^	0.86 (6)	2.57 (7)	3.239 (18)	136 (7)
O5—H5⋯O14*A* ^vi^	0.87 (6)	1.86 (6)	2.727 (9)	177 (8)
O5—H5⋯O14*B* ^vi^	0.87 (6)	1.93 (6)	2.763 (17)	162 (7)
O6—H6⋯O13*B*	0.86 (6)	2.45 (8)	2.98 (2)	120 (7)
O6—H6⋯O14*B* ^iv^	0.86 (6)	2.47 (7)	3.211 (17)	145 (7)
O7—H7*A*⋯O13*B* ^iv^	0.89 (3)	2.64 (4)	3.489 (18)	161 (7)
O7—H7*A*⋯O14*B* ^iv^	0.89 (3)	2.31 (6)	3.034 (19)	139 (7)

Symmetry codes: (i) 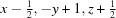; (ii) 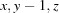; (iii) 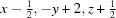; (iv) 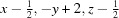; (v) 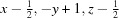; (vi) 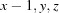.

**Table 2:** Experimental details

Crystal data	
Chemical formula	Cu_4_(SO)_4_(OH)_6_(H_2_O)
*M*_r_	470.28
Crystal system, space group	Monoclinic, *P* *n*
Temperature (K)	100
*a*, *b*, *c* (Å)	7.8400 (16), 6.3400 (13), 9.768 (2)
β (°)	107.32 (3)
*V* (Å^3^)	463.51 (18)
*Z*	2
Radiation type	Synchrotron, λ = 0.71074 Å
μ (mm^−1^)	9.33
Crystal size (mm)	0.15 × 0.10 × 0.05

Data collection	
Diffractometer	MX1 Beamline Australian Synchrotron
Absorption correction	Multi-scan (*SADABS*; Bruker, 2001 ▸)
*T*_min_, *T*_max_	0.517, 0.746
No. of measured, independent and observed [*I* > 2σ(*I*)] reflections	5709, 1763, 1652
*R*_int_	0.019
(sin θ/λ)_max_ (Å^−1^)	0.617

Refinement	
*R*[*F* ^2^ > 2σ(*F* ^2^)], *wR*(*F* ^2^), *S*	0.029, 0.074, 1.06
No. of reflections	1763
No. of parameters	157
No. of restraints	19
H-atom treatment	Only H-atom coordinates refined
Δρ_max_, Δρ_min_ (e Å^−3^)	0.67, −0.92
Absolute structure	Flack *x* determined using 745 quotients [(*I* ^+^)−(*I* ^−^)]/[(*I* ^+^)+(*I* ^−^)] (Parsons *et al.*, 2013 ▸)
Absolute structure parameter	0.128 (16)

Computer programs: *AS QEGUI*, *XDS* (Kabsch, 2010 ▸), *SHELXT* (Sheldrick, 2015*a* ▸), *SHELXL* (Sheldrick, 2015*b* ▸), *shelXle* (Hübschle *et al.*, 2011 ▸), *ORTEP-3 for Windows* (Farrugia, 2012 ▸) and *publCIF* (Westrip, 2010 ▸).

## supplementary crystallographic information

### Crystal data

**Table:**

Cu_4_(SO)_4_(OH)_6_(H_2_O)	*F*(000) = 456
*M**_r_* = 470.28	*D*_x_ = 3.370 Mg m^−^^3^
Monoclinic, *P**n*	Synchrotron radiation, λ = 0.71074 Å
*a* = 7.8400 (16) Å	Cell parameters from 96 reflections
*b* = 6.3400 (13) Å	µ = 9.33 mm^−^^1^
*c* = 9.768 (2) Å	*T* = 100 K
β = 107.32 (3)°	Plate, blue
*V* = 463.51 (18) Å^3^	0.15 × 0.10 × 0.05 mm
*Z* = 2	

### Data collection

**Table:**

MX1 Beamline Australian Synchrotron diffractometer	1652 reflections with *I* > 2σ(*I*)
Radiation source: Double Crystal Monochromator	*R*_int_ = 0.019
Silicon 111 scans	θ_max_ = 26.0°, θ_min_ = 2.9°
Absorption correction: multi-scan (SADABS; Bruker, 2001)	*h* = −9→9
*T*_min_ = 0.517, *T*_max_ = 0.746	*k* = −7→7
5709 measured reflections	*l* = −12→11
1763 independent reflections	

### Refinement

**Table:**

Refinement on *F*^2^	Hydrogen site location: difference Fourier map
Least-squares matrix: full	Only H-atom coordinates refined
*R*[*F*^2^ > 2σ(*F*^2^)] = 0.029	*w* = 1/[σ^2^(*F*_o_^2^) + (0.0447*P*)^2^] where *P* = (*F*_o_^2^ + 2*F*_c_^2^)/3
*wR*(*F*^2^) = 0.074	(Δ/σ)_max_ < 0.001
*S* = 1.06	Δρ_max_ = 0.67 e Å^−^^3^
1763 reflections	Δρ_min_ = −0.92 e Å^−^^3^
157 parameters	Absolute structure: Flack *x* determined using 745 quotients [(*I*^+^)-(*I*^-^)]/[(*I*^+^)+(*I*^-^)] (Parsons *et al.*, 2013)
19 restraints	Absolute structure parameter: 0.128 (16)

### Special details

**Table:**

Geometry. All esds (except the esd in the dihedral angle between two l.s. planes) are estimated using the full covariance matrix. The cell esds are taken into account individually in the estimation of esds in distances, angles and torsion angles; correlations between esds in cell parameters are only used when they are defined by crystal symmetry. An approximate (isotropic) treatment of cell esds is used for estimating esds involving l.s. planes.

### Fractional atomic coordinates and isotropic or equivalent isotropic displacement parameters (Å^2^)

**Table:**

	*x*	*y*	*z*	*U*_iso_*/*U*_eq_	Occ. (<1)
Cu1	0.64047 (12)	0.74631 (16)	0.76709 (10)	0.0112 (2)	
Cu2	0.40646 (10)	0.48699 (12)	0.51341 (8)	0.0113 (4)	
Cu3	0.37581 (9)	0.98271 (12)	0.52445 (7)	0.0117 (4)	
Cu4	0.14420 (12)	0.75064 (16)	0.26663 (9)	0.0108 (2)	
O1	0.4814 (10)	0.4981 (7)	0.7249 (8)	0.0150 (14)	
H1	0.398 (10)	0.481 (12)	0.765 (10)	0.022*	
O2	0.5487 (8)	0.2232 (8)	0.5561 (7)	0.0129 (11)	
H2	0.625 (9)	0.211 (12)	0.502 (8)	0.019*	
O3	0.4523 (10)	0.9538 (8)	0.7302 (7)	0.0157 (12)	
H3	0.382 (10)	0.941 (14)	0.779 (8)	0.024*	
O4	0.3320 (10)	0.4581 (8)	0.3087 (7)	0.0132 (12)	
H4	0.408 (10)	0.436 (12)	0.263 (8)	0.020*	
O5	0.2300 (9)	0.7197 (7)	0.4785 (7)	0.0145 (11)	
H5	0.134 (9)	0.715 (12)	0.505 (9)	0.022*	
O6	0.3032 (10)	1.0043 (7)	0.3128 (7)	0.0124 (13)	
H6	0.376 (10)	1.021 (11)	0.262 (9)	0.019*	
O11	0.6098 (9)	0.7456 (7)	0.5254 (8)	0.0170 (12)*	
O12	0.7217 (8)	0.6493 (7)	0.3281 (6)	0.0221 (10)*	
S1A	0.7607 (9)	0.7811 (8)	0.4618 (7)	0.0195 (19)*	0.7
O13A	0.7641 (12)	1.0103 (9)	0.4267 (9)	0.0178 (16)*	0.7
O14A	0.9297 (11)	0.7171 (13)	0.5649 (9)	0.0204 (17)*	0.7
S1B	0.7426 (14)	0.7916 (17)	0.4462 (11)	0.009 (3)*	0.3
O13B	0.699 (2)	1.008 (2)	0.3789 (19)	0.019 (4)*	0.3
O14B	0.922 (2)	0.799 (3)	0.5522 (17)	0.018 (4)*	0.3
O7	0.6288 (8)	0.7998 (8)	0.0410 (7)	0.0207 (10)	
H7B	0.645 (10)	0.722 (10)	0.117 (6)	0.031*	
H7A	0.526 (6)	0.869 (11)	0.020 (8)	0.031*	

### Atomic displacement parameters (Å^2^)

**Table:**

	*U*^11^	*U*^22^	*U*^33^	*U*^12^	*U*^13^	*U*^23^
Cu1	0.0116 (4)	0.0092 (4)	0.0113 (4)	0.0003 (2)	0.0010 (3)	0.0002 (2)
Cu2	0.0131 (9)	0.0097 (4)	0.0097 (6)	0.0000 (4)	0.0012 (5)	0.0004 (4)
Cu3	0.0124 (9)	0.0108 (4)	0.0098 (6)	0.0000 (5)	0.0002 (5)	−0.0005 (5)
Cu4	0.0124 (4)	0.0094 (4)	0.0094 (4)	−0.0011 (2)	0.0011 (3)	0.0001 (2)
O1	0.016 (4)	0.016 (2)	0.013 (3)	−0.0010 (19)	0.005 (2)	0.0006 (16)
O2	0.013 (3)	0.014 (3)	0.011 (2)	0.001 (2)	0.002 (2)	−0.0001 (18)
O3	0.017 (3)	0.018 (3)	0.013 (3)	0.000 (2)	0.006 (2)	0.000 (2)
O4	0.015 (3)	0.014 (3)	0.010 (3)	0.001 (2)	0.003 (2)	−0.001 (2)
O5	0.016 (3)	0.015 (2)	0.014 (2)	−0.001 (2)	0.006 (2)	0.000 (2)
O6	0.010 (3)	0.014 (3)	0.013 (3)	−0.0005 (18)	0.003 (2)	0.0006 (17)
O7	0.018 (2)	0.021 (3)	0.022 (3)	0.002 (2)	0.005 (2)	0.004 (2)

### Geometric parameters (Å, º)

**Table:**

Cu1—O3	1.929 (7)	Cu4—O5	1.986 (7)
Cu1—O4^i^	1.933 (7)	Cu4—O1^v^	1.993 (6)
Cu1—O1	1.974 (6)	Cu4—O6	2.002 (6)
Cu1—O6^ii^	1.997 (6)	Cu4—O4	2.327 (6)
Cu1—O11	2.300 (7)	Cu4—O3^vi^	2.363 (6)
Cu1—Cu3	3.0399 (14)	O1—H1	0.87 (6)
Cu1—Cu2^i^	3.0558 (14)	O2—H2	0.91 (6)
Cu2—O4	1.918 (7)	O3—H3	0.83 (6)
Cu2—O1	1.974 (7)	O4—H4	0.86 (6)
Cu2—O5	1.981 (6)	O5—H5	0.87 (6)
Cu2—O2	1.984 (5)	O6—H6	0.86 (6)
Cu2—O11	2.265 (6)	O11—S1B	1.499 (11)
Cu2—Cu4^i^	3.0194 (14)	O11—S1A	1.508 (8)
Cu3—O3	1.927 (7)	O12—S1B	1.435 (11)
Cu3—O6	1.979 (7)	O12—S1A	1.503 (6)
Cu3—O5	1.996 (6)	S1A—O14A	1.463 (9)
Cu3—O2^iii^	2.002 (6)	S1A—O13A	1.495 (8)
Cu3—O11	2.370 (6)	S1B—O14B	1.479 (17)
Cu3—O7^iv^	2.422 (6)	S1B—O13B	1.516 (19)
Cu3—Cu4	3.0135 (14)	O7—H7B	0.87 (3)
Cu4—O2^v^	1.975 (7)	O7—H7A	0.89 (3)
			
O3—Cu1—O4^i^	178.6 (4)	O6—Cu4—O4	106.4 (3)
O3—Cu1—O1	96.0 (3)	O2^v^—Cu4—O3^vi^	75.5 (2)
O4^i^—Cu1—O1	85.0 (3)	O5—Cu4—O3^vi^	103.8 (2)
O3—Cu1—O6^ii^	84.5 (3)	O1^v^—Cu4—O3^vi^	104.9 (3)
O4^i^—Cu1—O6^ii^	94.5 (3)	O6—Cu4—O3^vi^	73.9 (2)
O1—Cu1—O6^ii^	179.1 (4)	O4—Cu4—O3^vi^	178.4 (3)
O3—Cu1—O11	88.2 (3)	O2^v^—Cu4—Cu3	140.94 (16)
O4^i^—Cu1—O11	93.0 (2)	O5—Cu4—Cu3	40.93 (15)
O1—Cu1—O11	85.3 (3)	O1^v^—Cu4—Cu3	138.3 (2)
O6^ii^—Cu1—O11	95.5 (2)	O6—Cu4—Cu3	40.5 (2)
O3—Cu1—Cu3	37.9 (2)	O4—Cu4—Cu3	92.84 (17)
O4^i^—Cu1—Cu3	143.3 (2)	O3^vi^—Cu4—Cu3	86.39 (18)
O1—Cu1—Cu3	89.3 (2)	O2^v^—Cu4—Cu2^v^	40.42 (15)
O6^ii^—Cu1—Cu3	91.51 (19)	O5—Cu4—Cu2^v^	137.68 (16)
O11—Cu1—Cu3	50.39 (16)	O1^v^—Cu4—Cu2^v^	40.2 (2)
O3—Cu1—Cu2^i^	141.5 (2)	O6—Cu4—Cu2^v^	140.9 (2)
O4^i^—Cu1—Cu2^i^	37.3 (2)	O4—Cu4—Cu2^v^	87.18 (17)
O1—Cu1—Cu2^i^	91.1 (2)	O3^vi^—Cu4—Cu2^v^	93.56 (17)
O6^ii^—Cu1—Cu2^i^	88.07 (19)	Cu3—Cu4—Cu2^v^	178.43 (5)
O11—Cu1—Cu2^i^	130.19 (16)	Cu1—O1—Cu2	102.7 (3)
Cu3—Cu1—Cu2^i^	179.32 (4)	Cu1—O1—Cu4^i^	105.2 (4)
O4—Cu2—O1	176.5 (2)	Cu2—O1—Cu4^i^	99.1 (3)
O4—Cu2—O5	84.9 (3)	Cu1—O1—H1	122 (5)
O1—Cu2—O5	97.4 (3)	Cu2—O1—H1	116 (6)
O4—Cu2—O2	96.7 (2)	Cu4^i^—O1—H1	110 (5)
O1—Cu2—O2	80.6 (3)	Cu4^i^—O2—Cu2	99.4 (2)
O5—Cu2—O2	169.6 (3)	Cu4^i^—O2—Cu3^vii^	104.7 (3)
O4—Cu2—O11	96.6 (2)	Cu2—O2—Cu3^vii^	107.3 (3)
O1—Cu2—O11	86.2 (3)	Cu4^i^—O2—H2	120 (5)
O5—Cu2—O11	85.1 (2)	Cu2—O2—H2	112 (5)
O2—Cu2—O11	104.8 (3)	Cu3^vii^—O2—H2	112 (5)
O4—Cu2—Cu4^i^	136.77 (17)	Cu3—O3—Cu1	104.1 (3)
O1—Cu2—Cu4^i^	40.67 (17)	Cu3—O3—Cu4^ii^	93.9 (2)
O5—Cu2—Cu4^i^	137.9 (2)	Cu1—O3—Cu4^ii^	95.5 (3)
O2—Cu2—Cu4^i^	40.18 (19)	Cu3—O3—H3	124 (6)
O11—Cu2—Cu4^i^	93.75 (18)	Cu1—O3—H3	115 (6)
O4—Cu2—Cu1^v^	37.7 (2)	Cu4^ii^—O3—H3	119 (6)
O1—Cu2—Cu1^v^	139.7 (2)	Cu2—O4—Cu1^v^	105.0 (3)
O5—Cu2—Cu1^v^	86.97 (19)	Cu2—O4—Cu4	95.1 (2)
O2—Cu2—Cu1^v^	88.10 (19)	Cu1^v^—O4—Cu4	94.9 (3)
O11—Cu2—Cu1^v^	134.10 (19)	Cu2—O4—H4	121 (5)
Cu4^i^—Cu2—Cu1^v^	120.91 (4)	Cu1^v^—O4—H4	113 (5)
O3—Cu3—O6	178.0 (3)	Cu4—O4—H4	123 (5)
O3—Cu3—O5	97.7 (2)	Cu2—O5—Cu4	104.8 (3)
O6—Cu3—O5	81.7 (3)	Cu2—O5—Cu3	105.1 (3)
O3—Cu3—O2^iii^	85.7 (3)	Cu4—O5—Cu3	98.4 (2)
O6—Cu3—O2^iii^	94.6 (2)	Cu2—O5—H5	123 (5)
O5—Cu3—O2^iii^	170.8 (3)	Cu4—O5—H5	105 (6)
O3—Cu3—O11	86.3 (3)	Cu3—O5—H5	117 (5)
O6—Cu3—O11	91.7 (2)	Cu3—O6—Cu1^vi^	104.7 (3)
O5—Cu3—O11	82.0 (2)	Cu3—O6—Cu4	98.4 (3)
O2^iii^—Cu3—O11	89.7 (2)	Cu1^vi^—O6—Cu4	105.8 (4)
O3—Cu3—O7^iv^	89.5 (2)	Cu3—O6—H6	125 (6)
O6—Cu3—O7^iv^	92.5 (3)	Cu1^vi^—O6—H6	105 (5)
O5—Cu3—O7^iv^	94.3 (2)	Cu4—O6—H6	116 (5)
O2^iii^—Cu3—O7^iv^	94.3 (2)	S1B—O11—Cu2	134.4 (5)
O11—Cu3—O7^iv^	174.0 (2)	S1A—O11—Cu2	135.5 (4)
O3—Cu3—Cu4	138.42 (17)	S1B—O11—Cu1	130.7 (5)
O6—Cu3—Cu4	41.10 (16)	S1A—O11—Cu1	124.6 (4)
O5—Cu3—Cu4	40.70 (19)	Cu2—O11—Cu1	84.9 (2)
O2^iii^—Cu3—Cu4	135.46 (18)	S1B—O11—Cu3	122.0 (5)
O11—Cu3—Cu4	88.09 (17)	S1A—O11—Cu3	127.2 (3)
O7^iv^—Cu3—Cu4	92.24 (15)	Cu2—O11—Cu3	85.8 (2)
O3—Cu3—Cu1	37.98 (19)	Cu1—O11—Cu3	81.2 (2)
O6—Cu3—Cu1	139.99 (19)	O14A—S1A—O13A	110.7 (6)
O5—Cu3—Cu1	88.6 (2)	O14A—S1A—O12	110.6 (5)
O2^iii^—Cu3—Cu1	88.94 (17)	O13A—S1A—O12	110.8 (5)
O11—Cu3—Cu1	48.41 (18)	O14A—S1A—O11	109.6 (5)
O7^iv^—Cu3—Cu1	127.04 (15)	O13A—S1A—O11	108.0 (5)
Cu4—Cu3—Cu1	120.83 (4)	O12—S1A—O11	107.1 (4)
O2^v^—Cu4—O5	177.5 (3)	O12—S1B—O14B	116.1 (10)
O2^v^—Cu4—O1^v^	80.3 (3)	O12—S1B—O11	111.2 (7)
O5—Cu4—O1^v^	97.6 (3)	O14B—S1B—O11	107.9 (9)
O2^v^—Cu4—O6	100.6 (3)	O12—S1B—O13B	105.3 (9)
O5—Cu4—O6	81.3 (3)	O14B—S1B—O13B	108.6 (12)
O1^v^—Cu4—O6	178.1 (4)	O11—S1B—O13B	107.3 (10)
O2^v^—Cu4—O4	105.9 (2)	Cu3^viii^—O7—H7B	118 (5)
O5—Cu4—O4	74.8 (2)	Cu3^viii^—O7—H7A	113 (5)
O1^v^—Cu4—O4	74.8 (2)	H7B—O7—H7A	112 (6)

Symmetry codes: (i) *x*+1/2, −*y*+1, *z*+1/2; (ii) *x*+1/2, −*y*+2, *z*+1/2; (iii) *x*, *y*+1, *z*; (iv) *x*−1/2, −*y*+2, *z*+1/2; (v) *x*−1/2, −*y*+1, *z*−1/2; (vi) *x*−1/2, −*y*+2, *z*−1/2; (vii) *x*, *y*−1, *z*; (viii) *x*+1/2, −*y*+2, *z*−1/2.

### Hydrogen-bond geometry (Å, º)

**Table:**

*D*—H···*A*	*D*—H	H···*A*	*D*···*A*	*D*—H···*A*
O1—H1···O12^ix^	0.87 (6)	1.86 (6)	2.693 (9)	160 (7)
O7—H7*B*···O12	0.87 (3)	2.02 (4)	2.844 (7)	158 (7)
O2—H2···O13*A*^vii^	0.91 (6)	1.95 (6)	2.746 (9)	144 (7)
O2—H2···O13*B*^vii^	0.91 (6)	1.96 (7)	2.731 (18)	141 (7)
O3—H3···O13*A*^iv^	0.83 (6)	1.96 (6)	2.757 (10)	161 (9)
O3—H3···O13*B*^iv^	0.83 (6)	1.99 (7)	2.799 (19)	164 (9)
O7—H7*A*···O13*A*^vi^	0.89 (3)	2.13 (3)	2.995 (10)	167 (7)
O4—H4···O14*A*^v^	0.86 (6)	2.22 (7)	2.929 (9)	141 (7)
O4—H4···O14*B*^v^	0.86 (6)	2.57 (7)	3.239 (18)	136 (7)
O5—H5···O14*A*^x^	0.87 (6)	1.86 (6)	2.727 (9)	177 (8)
O5—H5···O14*B*^x^	0.87 (6)	1.93 (6)	2.763 (17)	162 (7)
O6—H6···O13*B*	0.86 (6)	2.45 (8)	2.98 (2)	120 (7)
O6—H6···O14*B*^vi^	0.86 (6)	2.47 (7)	3.211 (17)	145 (7)
O7—H7*A*···O13*B*^vi^	0.89 (3)	2.64 (4)	3.489 (18)	161 (7)
O7—H7*A*···O14*B*^vi^	0.89 (3)	2.31 (6)	3.034 (19)	139 (7)

Symmetry codes: (iv) *x*−1/2, −*y*+2, *z*+1/2; (v) *x*−1/2, −*y*+1, *z*−1/2; (vi) *x*−1/2, −*y*+2, *z*−1/2; (vii) *x*, *y*−1, *z*; (ix) *x*−1/2, −*y*+1, *z*+1/2; (x) *x*−1, *y*, *z*.
